# Cognitive-Emotional Involvement During Mirror Exposure Is Not Accompanied by Physiological Activation in Binge Eating Disorder

**DOI:** 10.3389/fpsyt.2019.00745

**Published:** 2019-11-19

**Authors:** Julia Baur, Kerstin Krohmer, Brunna Tuschen-Caffier, Jennifer Svaldi

**Affiliations:** ^1^Department of Clinical Psychology and Psychotherapy, University of Tuebingen, Tuebingen, Germany; ^2^Department of Clinical Psychology and Psychotherapy, University of Freiburg, Freiburg, Germany

**Keywords:** binge eating disorder, body image, mirror exposure, fundamental frequency, physiology

## Abstract

Body image interventions have been shown to reduce self-reported cognitive-emotional facets of body image disturbance in binge eating disorder (BED). However, more objective assessment methods are required to evaluate the effects of these interventions. Therefore, the present study aimed at investigating the usefulness of vocally encoded emotional arousal as physiological correlate of body dissatisfaction during mirror exposure in women with BED. Women with BED (*n* = 60) and weight-matched controls (CG; *n* = 60) participated in an experimental thought-sampling procedure including a mirror exposure and a control condition in a repeated-measures design. Fundamental frequency as a vocal correlate of emotional arousal as well as negative, neutral, and positive body-related cognitions during both conditions were analyzed. In line with our hypotheses, the BED group verbalized more negative, and less positive and neutral body-related cognitions during the mirror exposure condition compared to the CG. Contrary to our hypotheses, though, there was a stronger *in*crease in physiological arousal between the control and the mirror exposure condition in the CG relative to the BED group. Furthermore, a significant negative correlation between fundamental frequency and the severity of cognitive-emotional body image disturbances emerged. The findings indicate a cognitive-emotional over-involvement with physical appearance during mirror exposure in women with BED compared to weight-matched controls in the absence of a corresponding physiological pattern. Results are discussed in terms of an impaired ability of women with BED to show adequate physiological responses to body-related stress. In addition, methodological recommendations for future research are presented.

## Introduction

According to the Diagnostic and Statistical Manual of Mental Disorders (1) binge eating disorder (BED) is characterized by recurrent episodes of binge eating accompanied by feelings of loss of control in the absence of inappropriate compensatory weight regulation behavior as in bulimia nervosa (BN). BED is the most prevalent eating disorder (2) and is associated with overweight and obesity, elevated psychological and medical comorbidity, as well as psychosocial impairment. These severe consequences illustrate the need to identify and understand the core mechanisms underlying this eating disorder (3–5).

Although the diagnostic criteria for BED do not include a criterion relating to body image disturbances, there is increasing empirical evidence for the detrimental influence of a negative body image in patients with BED (for an overview see 6). Body image is a multifaceted construct, which consists of a perceptual, a cognitive-affective and a behavioral component (7). The cognitive-affective component, which comprises attitudes, cognitions, and emotions regarding one’s own body (e.g., body dissatisfaction, overvaluation of shape and weight), seems to be especially relevant in distinguishing patients with BED from weight-matched individuals without an eating disorder. These results underline the assumption that body image disturbances in women with BED are not only a result of increased weight, but of eating pathology (8, 9). This assumption is furthermore supported by the fact that the level of body image disturbances observed in BED is comparable to the level of impairment observed in patients with BN (10). Besides being linked to severe functional impairment, eating disorder psychopathology, and depression in women with BED (11, 12), numerous studies emphasize the importance of body image disturbances for the development and maintenance of BED. As such, longitudinal studies have identified body dissatisfaction as a risk factor for the development of BED in adolescent females (e.g., 13, 14). Furthermore, negative mood preceding binge episodes is most frequently triggered by weight and shape related issues (15). This corroborates findings from an experimental study in which desire to binge and salivation during mirror exposure relative to a control condition were only increased in women with BED but not in weight-matched controls (16). Beyond that, overvaluation of shape and weight has demonstrated to be one of the most salient predictors and moderators for remission rates in the treatment of BED. Thereby, the presence of overvaluation of shape and weight was associated with significantly lower remission rates at the end of both a pharmacological and a cognitive-behavioral treatment (CBT) (17). Additionally, elevated levels of weight concern also negatively influenced remission rates during a 4-year follow-up period following CBT treatment in another study (18).

Mirror exposure has been shown to be a validated treatment for the improvement of body image disturbances in patients with clinical and subclinical eating disorders (19). It encompasses the systematic, repetitive viewing of oneself in a mirror under therapeutic guidance mostly including a non-judgmental description of the own body parts (19–22). In BED, two studies have shown that mirror exposure leads to significant improvements in the cognitive-affective component of body image, as well as to substantial and stable improvements in eating-disorder specific and overall psychopathology (23, 24). Finally, in one study (25), participants with BED underwent a prolonged and repeated experimental mirror exposure task without therapeutic support. Here, a decrease in negative mood and negative body-related cognitions as well as an increase in appearance self-esteem was found after two prolonged mirror exposures. This corroborates the assumption that one possible underlying mechanism of change during mirror exposure might be habituation to the discomfort and negative affect elucidated through the confrontation with one’s own body (for a review see 19). Of note, though, the reported cognitive-emotional habituation effects in mirror exposure mostly rely on self-report data (22, 26, 27) and little research so far has focused on more objective, psychophysiological responses during mirror exposure in patients with BED. A more objective approach though is important as self-report measures are prone to social desirability biases which might be especially relevant in treatment evaluation (28). Second, as habituation is said to be one of the mechanisms responsible for improvements following mirror exposure therapy (19), research on physiological activation during mirror exposure warrants further attention.

Admittedly, though, results concerning changes in physiological arousal over the course of mirror exposure as measured by heart rate (variability), skin conductance, and salivary cortisol have been inconclusive despite concomitantly obtained group differences in self-reported cognitive-affective body image disturbances (29–32). For example, in the study conducted by Servián-Franco et al. (31), a greater level of arousal while focusing on thinness-related body parts like hips, buttocks, and abdomen during mirror exposure was found in both high and low body-dissatisfied women, whereas significantly higher levels of negative emotions and cognitions were observed in highly dissatisfied women compared to controls. Two studies investigating physiological changes following body image therapy also reported heterogeneous results. While Trentowska et al. (32) found no changes in psychophysiology in women with BN using heart rate (variability) and skin conductance level prior to and after mirror exposure therapy, neuroendocrine changes measured by cortisol level were found in a sample with patients with BN in a study conducted by Díaz-Ferrer et al. (30). In BED, only one study so far has investigated the effect of a mirror exposure by using both self-reported and physiological measures of stress response. While Naumann et al. (33) found women with BED and weight-matched controls to show an increase in body dissatisfaction after mirror exposure compared to no mirror exposure following a socioevaluative stress induction by means of the Trier Social Stress Test for Groups (TSST-G), this increase was significantly more pronounced in women with BED. However, no difference in physiological arousal measured by salivary cortisol was found. Given the inconclusive results concerning physiological activation during mirror exposure, further research is needed to understand the relationship between the cognitive-affective and the physiological activation during mirror exposure. Notably, physiological assessment methods used in previous studies have been criticized due to their invasiveness and visibility during mirror exposure potentially serving as visible distractor (32, 34). A recently-discussed, non-invasive way of measuring emotional arousal is the assessment of vocal fundamental frequency (35).

Fundamental frequency (f0) refers to the vibration of the vocal folds during phonation and is highly correlated with perceived pitch. During stress, higher f0 scores have been observed in simulated as well as in naturally occurring stressful situations due to the heightened tension in the involved speech muscles (36). In psychological research, f0 has already been used as a correlate of emotional arousal in research on couple therapy as well as in research on mental disorders, especially anxiety disorders (e.g., 37–42). Referring to some recent results, f0 was able to differentiate between persons with and without the diagnosis of a social anxiety disorder during a stress-provoking speech task (38). In a study on couple interactions, significant correlations between f0 and other physiological measures like heart rate variability, blood pressure, and cortisol were found, further underpinning the usefulness of f0 in psychological research (39). In the context of body dissatisfaction, only one study so far has proven the utility of vocally encoded emotional arousal as a physiological correlate of body distress. During an experimental mirror exposure, vocally encoded emotional arousal was able to differentiate between women with overweight and obesity and normal weight controls, and correlated significantly with different validated measures of body image (43). Furthermore, encouraging results have been found concerning the predictive value of f0 in treatment studies. Vocally encoded emotional arousal measured during a stress-provoking task prior to and after pharmacotherapy was able to distinguish between treatment-responder and non-responder in patients with social phobia; similarly, in couple therapy, treatment was predicted by spouses’ f0 scores during a couple conflict prior to therapy (34, 44). Against this backdrop, vocally encoded emotional arousal seems to be a convincing alternative for assessing emotional arousal during a stress-provoking task in mental disorders.

Thus, this study aimed at investigating the psychophysiological and cognitive-affective reactions to an experimental mirror exposure in women with BED using vocally encoded emotional arousal during a thought-sampling procedure. We hypothesized that women with BED would show a greater increase in f0 between the control and the mirror exposure condition compared to weight-matched women without an eating disorder. In line with previous studies (e.g., 23, 45), we expected more negative (and fewer positive) body-related cognitions and more self-reported negative emotions by means of visual analogue scales (stress, insecurity, body dissatisfaction) in the BED group compared to controls during the mirror exposure relative to the control condition. Furthermore, significant positive correlations between the difference score of f0 between the two experimental conditions, and validated state and trait measure of body image were hypothesized.

## Materials and Methods

### Participants

The study was approved by the Ethics Committee of the Medical Faculty of the University of Tuebingen (575/2014BO2). Females with binge eating disorder (BED; *n* = 60) and females with overweight and obesity without an eating disorder (CG; *n* = 60) were eligible for participating in this study. The BED sample was recruited from an ongoing randomized controlled trial on therapeutic mirror exposure in BED (data from the comparative RCT will be presented elsewhere). The present study was conducted as part of a baseline assessment prior to randomization to the RCT. The CG was matched to the BED group on BMI and age.

Inclusion criteria for both groups were a) age between 18 and 69, b) female gender, c) corrected or normal vision, and d) German language skills. Furthermore the CG had to have a body mass index (BMI) of > = 25 and no lifetime diagnosis of an eating disorder. Exclusion criteria for both groups consisted of the presence of a) acute psychosis, severe suicidal ideation, manic episode, or substance abuse/addiction, b) pregnancy or lactation period, c) borderline personality disorder, or d) severe physical illness. Eating disorder pathology and the present diagnosis of BED according to DSM-V (1 binge/week during the last 3 months) was assessed using the Eating Disorder Examination (EDE; German version: 46) while all other mental disorders were screened by the Structured Clinical Interview (SCID) for DSM-IV Axis I and Axis II Disorders (German version: 47). All participants were financially rewarded.

There were no differences between the two groups regarding age, BMI, and educational level (see **Table 1**). However, women with BED were less frequently in a partnership than controls. As expected, the BED group self-reported higher scores on measures of eating pathology and severity of depression. In line with previous research (3) comorbid mental disorders were more frequent in the BED relative to the CG, whereby anxiety and affective disorders were the most frequent ones (anxiety disorders: BED: 18.6%/ CG: 6.8%; affective disorders: BED: 10.2%/ CG: 3.4%).

**Table 1:** Descriptive characteristics of demographic and psychopathological variables for women with binge eating disorder (BED) and women with overweight and obesity (CG).

	BED (*n* = 60)	CG (*n* = 60)	Statistics
	*Frequency*	*Frequency*	
Education level^a^			
low	16	18	χ*^2^* (*1*) = .165
high	43	41	
Marital status^a^			χ*^2^* (*2*) = 6.995*
with partner	27	41	
single	23	14	
widowed/divorced	9	4	
Comorbid diagnosis^a^	16	7	χ*^2^* (*1*) = 4.180*
	***M (SD)***	***M (SD)***	
Age (years)	42.2 (14.6)	40.13 (14.8)	*F*(1,118) = 0.575
BMI	32.8 (6.0)	30.9 (9.0)	*F*(1,118) = 1.868
BDI	18.9 (11.8)	7.8 (7.6)	*F*(1,118) = 37.404**
EDE_global_	2.4 (0.9)	1.4 (0.9)	*F*(1,118) = 43.596**
EDE_shape concerns_	3.6 (1.1)	2.1 (1.2)	*F*(1,118) = 55.750**
EDE_weight concerns_	3.2 (1.2)	1.9 (1.2)	*F*(1,118) = 32.332**
EDE_restraint eating_	1.4 (1.3)	0.9 (1.1)	*F*(1,118) = 4.736*
EDE_eating concerns_	1.5 (1.2)	0.5 (0.9)	*F*(1,118) = 25.243**
BSQ	126.6 (25.1)	87.9 (30.8)	*F*(1,118) = 56.916**

BDI, Beck Depression Inventory; BMI, Body Mass Index; BSQ, Body Shape Questionnaire; EDE, Eating Disorder Examination (for detailed description see the section Questionnaires and Interviews); educational level: low ≤ secondary school; high = baccalaureate or university degree.

* p < .05; ** p < .001.

^a^n = 59 in the BED group and the CG due to missing data in questionnaire assessment.

### Measures

#### Experimental Thought-Sampling

A thought-sampling task was implemented to assess the occurrence of negative, neutral, and positive body-related verbalizations as well as to measure vocally encoded emotional arousal. Therefore, participants were instructed to verbalize all their concurrent cognitions and emotions in two different conditions each lasting 5 min while standing alone in a small soundproof room.

During the control condition (CC), participants stood in front of a closed mirror wearing their street wear, while during mirror exposure (ME) they wore a standardized underwear (nude panty and top) while they were standing in front of a three-winged full-length mirror. A microphone placed on the ceiling of the room recorded all verbalizations. Before starting with the two experimental conditions, participants practiced thinking aloud while the investigator was present in order to familiarize with the task.

Each condition started with a 2-min relaxation task to direct participants’ attention on their concurrent feelings and thoughts. The order of the two conditions was randomized and counterbalanced between the two groups. At the beginning of the experiment as well as after each experimental condition, participants had to fill in some questionnaires measuring current stress, insecurity, and body satisfaction level as well as the motivation to follow the instructions (for further information, see section *Questionnaires and Interviews*). After the task, participants were debriefed.

#### Questionnaires and Interviews

1) Body dissatisfaction was assessed using the German Version of the *Body Shape Questionnaire* (BSQ; 48; German version: 49). The *BSQ* is a 34-item self-report questionnaire, which is widely used to assess weight and shape concerns over the past four weeks on a six-response scale for each item ranging from 1 (never) to 6 (always). The minimum score is 34, reflecting no shape and weight concerns, whereas the maximum score of 204 reflects extreme shape and weight concerns. The BSQ shows excellent reliability, high sensitivity, and validity (50). Internal consistency was α = .959 in our sample. 2) Severity of depressive symptoms over the last 2 weeks was measured by the German version of the *Beck Depression Inventory-II* (BDI-II; 51; German version: 52), a 21-item self-report questionnaire with a four-response scale ranging from 0 to 3, with higher scores indicating higher symptom severity. The maximum sum score is 63, with 0–13 representing no depressive symptoms, 14–19 mild, 20–28 moderate depressive, and more than 28 severe depressive symptoms. Several studies confirmed the BDI’s high internal consistency, reliability, and discriminant validity (53). 3) Three 10 cm visual analogue scales (VAS) anchored “not at all” (0) and “completely” (10) implemented prior to and at the end of each experimental condition were used to assess current feelings of insecurity, stress, and body satisfaction by means of the items “At the moment, how satisfied are you with your body?” and “At the moment, how insecure/stressed do you feel?.” Another 10 cm VAS anchored “*not at all*” (0) and “completely” (10) was used to assess the self-reported motivation to follow the instructions of thought-sampling by the item “Please evaluate your motivation to follow the instruction of thought-sampling.”

### Procedure

Participants were recruited via newspaper announcements, flyers in medical centers, and pharmacies as well as by e-mails of the local university. Interested participants were contacted for a short telephone screening to assess inclusion and exclusion criteria. Eligible participants were then invited to a face-to-face diagnostic assessment by trained psychologists at the local university to assess eating and general psychopathology by means of the EDE and SCID as wells as to fill in questionnaires on sociodemographic and (eating) psychopathological information via Unipark (Globalpark AG, Hürth), an online survey platform. Prior to the diagnostic assessment, participants were given a detailed study description and signed informed consent. An appointment for the experimental thought-sampling task was scheduled approximately one week after the diagnostic assessment. Participants then completed the above-mentioned experimental thought-sampling procedure.

### Data Reduction

*Quantitative content analysis:* All audio files were blinded and transcribed following prescribed rules (54) and then coded by independent raters by means of valence (negative vs. positive vs. neutral) and content (body-related vs. non body-related). For the present results, only body-related verbalizations were analyzed, being quantified as the percentage of all verbalized cognitions and emotions during each condition. Verbalizations were coded as body-related when participants talked about weight, shape, physical appearance, age, as well as body-related activities such as body care or fitness. All other cognitions were rated as non-body-related. Concerning valence, body-related cognitions were coded as positive when agreement or acceptance with the own body was verbalized (e.g., “I feel like I don’t have to hide my body.”), and as negative when participants talked depreciative about their body (e.g., “When I look at my stomach, I feel frustrated. It’s just too much!”). Verbalizations, which dealt with the participant’s former body or the appearance of other persons without direct comparison to their own body as well as all other body-related descriptive verbalizations, which were neither positive nor negative, were coded as neutral (e.g., “I have wide hips.”). To assess interrater-reliability, a second independent rater coded 40 randomly selected transcripts (10 transcripts of each condition of each group). Separated for contents of cognitions, raters on average agreed in 96.0% of the cases and on 89.8% concerning valence of cognitions. Overall, reliability was good with 78.3% between the two raters. As control variable, the percentage of time spoken during both conditions as well as the motivation to follow the instructions was measured.

*Vocally encoded emotional arousal:* For the voice analysis, the records were analyzed with Praat, a free multiple platform program for speech analysis (55). Before analyzing fundamental frequency, detection errors [background noise (e.g., closing of a window) and nonverbal speech sounds (sighing, coughing, etc.) as well as the detection of periodicity in unvoiced speech segments] were manually removed from each recording. No correction was applied when the algorithm was not able to detect periodicity in voiced segments (e.g., due to irregular phonation). Range was adapted by the method introduced by Daniel Hirst in order to restrict f0 scores to the normal range of adult speech (56). In line with previous research (e.g., 57), analysis started with the second minute of each condition. For each condition, mean f0 estimates were than obtained continuously from all voiced segments for each 25 ms using autocorrelation methods outlined by Praat, resulting in a mean f0 score per condition and person.

To account for individual differences in fundamental frequency, difference scores were calculated per person between the two conditions (Diff_f0_mirror-control_ = mean_f0^mirror^ - mean_f0^control^). Positive difference scores, therefore, indicate more emotional arousal during the mirror exposure relative to the control condition, whereas negative difference scores indicate less emotional arousal during the mirror exposure compared to the control condition (see **Tables 2** and **3** for descriptive statistics of the dependent variables).

**Table 2:** Means (M) and standard deviations (SD) for dependent variables for women with binge eating disorder (BED) and women with overweight and obesity (CG).

	BED (*n = 60)*		CG (*n = 60)*	
	*M (SD)*		*M (SD)*	
	ME	CC	ME	CC
% of time spoken	23.9 (12.0)	30.7 (12.5)	23.4 (13.0)	25.8 (13.3)
Motivation	3.60 (1.44)	3.71 (1.28)	3.71 (1.34)	3.63 (1.33)
Diff_f0_mirror-control_	5.7 (8.3)^a^		9.5 (9.7)	
*Body-related cognitions (BC)*				
negative	58.85 (16.49)	18.35 (19.05)	38.00 (20.27)	7.25 (11.18)
neutral	22.21 (13.00)	12.49 (12.27)	33.67 (15.64)	10.19 (14.67)
positive	4.36 (4.44)	0.68 (1.61)	9.18 (9.04)	1.00 (2.28)

CC, control condition; Diff_f0_mirror-control_, difference score of f0 between the mirror exposure and control condition; ME, mirror exposure condition; motivation, self-reported motivation to follow the instructions of thought-sampling; % of time spoken, percentage of time spent talking during each condition.

^a^n = 58 in the BED group due to the exclusion of two outliers.

**Table 3:** Means (M) and standard deviations (SD) for questionnaire assessment during thought-sampling for women with binge eating disorder (BED) and women with overweight and obesity (CG).

	BED (*n = 60)*			CG (*n = 60)*		
	*M (SD)*			*M (SD)*		
	Baseline	CC	ME	Baseline	CC	ME
*Visual analogue scales*						
stress	4.6 (2.5)	4.3 (2.5)	6.3 (2.7)	3.0 (2.5)	2.7 (2.4)	3.7 (2.6)
insecurity	4.9 (2.7)	4.3 (2.7)	6.7 (2.8)	3.1 (2.5)	2.7 (2.3)	4.2 (2.9)
body satisfaction	2.2 (1.8)	2.0 (1.9)	0.7 (0.9)	4.5 (2.0)	4.4 (2.5)	2.7 (2.6)

Baseline, beginning of experimental procedure; CC, control condition; ME, mirror exposure condition.

### Data Analysis

Statistical analyses were made using IBM SPSS Statistics (Version 25.2). We handled missing data by using mean substitution (body-related cognitions/f0: one participant per group due to technical problems in data storing; Interview assessment: one participant per group; BDI-II: three participants with BED; VAS during thought-sampling: five participants of the CG and one participant with BED). Box plot analysis outlined by SPSS were used to detect outliers, extreme outliers (> three times the interquartile range) were excluded from the analyses. Two participants in the BED group had to be excluded for the voice analysis and one participant had to be excluded concerning body satisfaction ratings during ME.

Hypotheses were tested by means of a univariate analysis of variance (ANOVA) for the vocal analysis and by repeated-measures ANOVA for body-related cognitions and visual analogue scales with condition as within-subject factor (mirror exposure vs. control condition (vs. baseline for VAS)) and group as between-subject factor (BED vs. CG). *Post hoc* ANOVAS and t-tests with Bonferroni correction for multiple testing were applied when significant main effects and interactions were detected. If assumption of sphericity was not met (Mauchlys Sphericity Test: p < 0.05), degrees of freedom for dependent variables were corrected conservatively by Greenhouse-Geisser. Being exceedingly robust against violation of normality (58), ANOVAs were also adopted for variables not normal in distribution. Pearson product-moment correlation coefficients were computed to assess the relationship between vocally encoded emotional arousal and body-related measures. Effect sizes of the group differences and interactions are reported by partial eta squared (*η*_p_²), whereby values larger than 0.01 refer to small, 0.06 to moderate, and 0.14 to large effect sizes (59).

## Results

### Manipulation Check

*Percentage of time spoken*: A 2 (Group: BED, CG) × 2 (Condition: ME, CC) repeated-measures ANOVA for the percentage of time spoken showed a significant main effect of Condition [*F*(1,118) = 32.928, *p* < .001, *η* *_p_* *²* = .218] as well as a significant interaction of Group × Condition [*F*(1,118) = 7.686, *p* = .006, *η* *_p_* *²* = .061], whereas the main effect of Group was not significant [*F*(1,118) = 1.581, *p* = .211, *η* *_p_* *²* = .013]. *Post hoc* tests showed that both groups verbalized more cognitions during CC than ME [BED: *F*(1,59) = 33.931, *p* < .001, *η* *_p_* *²* = .365; CG: *F*(1,59) = 4.716, *p* = .034, *η* *_p_* *²* = .074]. Compared to CG, women with BED verbalized more cognitions during CC [*F*(1,118) = 4.459, *p* = .037, *η* *_p_* *²* = .037], while no difference was found in the amount of verbalized cognitions in the ME [*F*(1,118) = .049, *p* = .825, *η* *_p_* *²* < .001].

*Motivation:* A 2 (Group: BED, CG) × 2 (Condition: ME, CC) repeated-measures ANOVA for the motivation to follow the instructions of thought-sampling showed no significant main effects or interaction [all *F*s(1,118) *≤* 1.654, *ps* ≥ .202, *η* *_p_* *² s* ≤ .014].

### Fundamental Frequency

**Figure 1:**
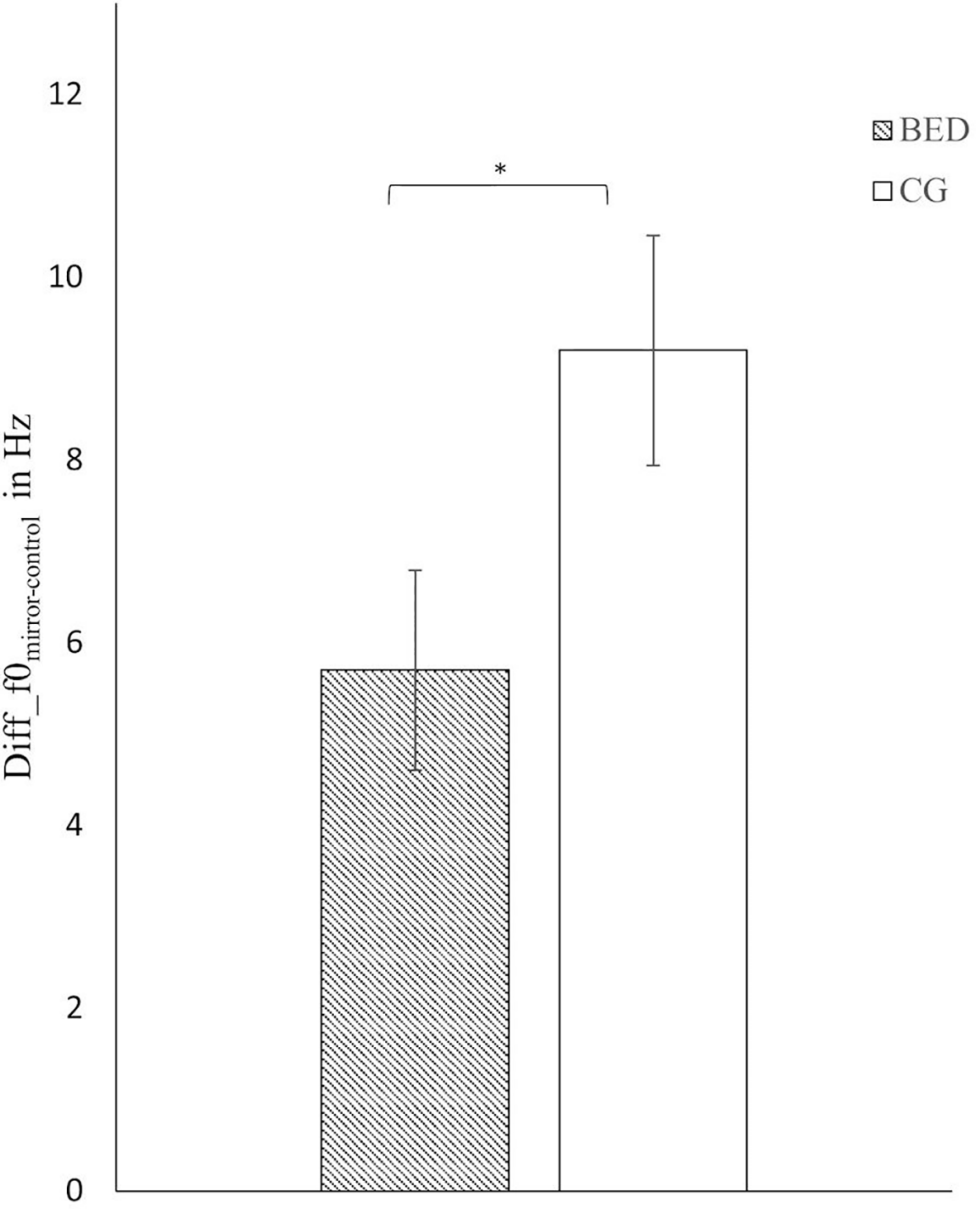
Mean and standard error for the difference score in mean f0 between the mirror exposure and control condition in the BED and the control group (CG). *p < .05.

Regarding f0, a significant main effect of Group was found [*F*(1,116) = 4.409, *p* = .038, *η* *_p_* *²* = .037] indicating a stronger increase in f0 from CC to ME in the CG compared to BED group (**Figure 1**).

### Valence of Body-Related Cognitions

**Figure 2:**
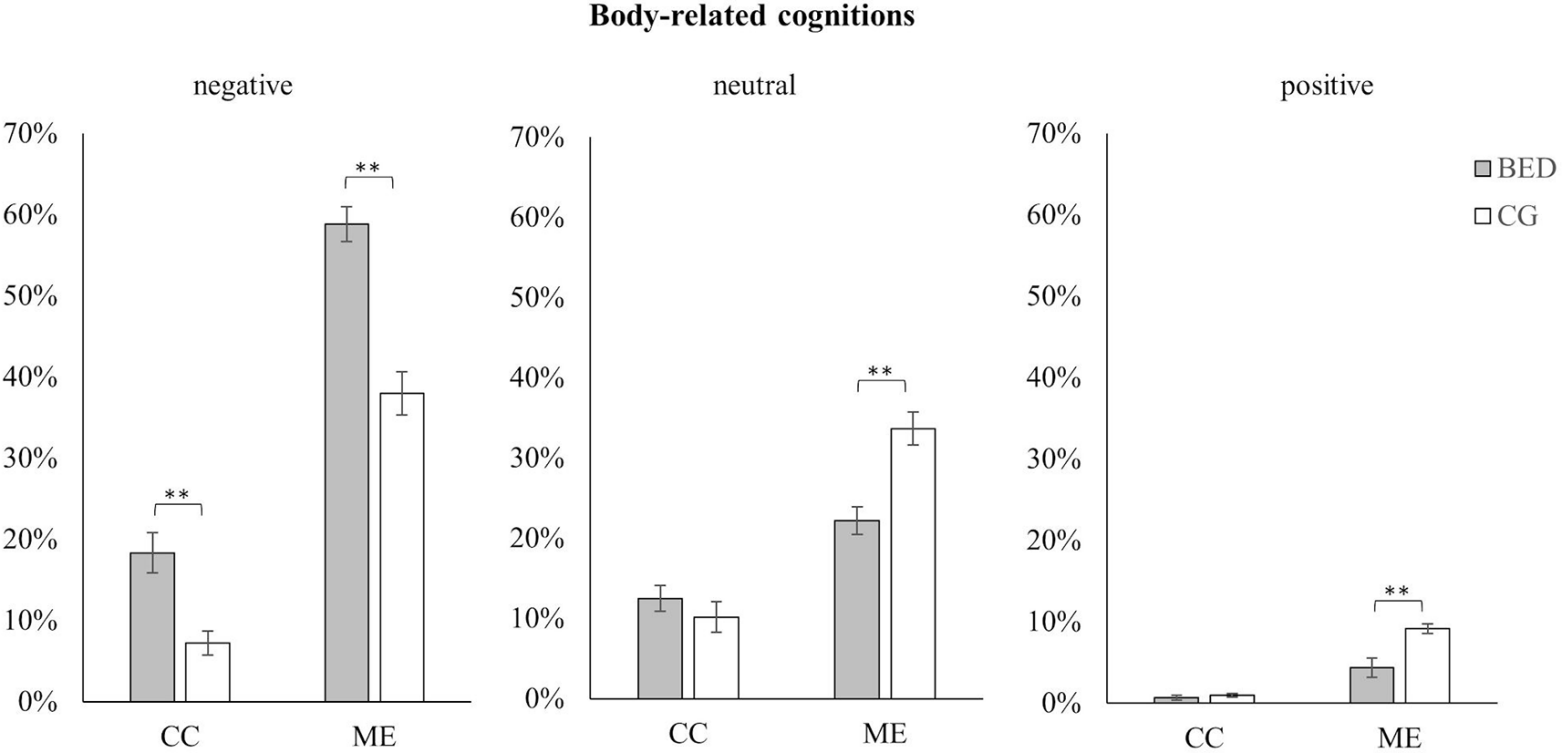
Mean and standard error for negative, neutral, and positive body-related cognitions in women with BED and the control group (CG) during the control (CC) and the mirror exposure condition (ME).** p < .01.

Regarding the valence of body-related cognitions, the three-way ANOVA for repeated measures (Group × Condition × Valence) revealed a significant three-way interaction (for statistics see **Table 4**). To address our hypotheses, we conducted *post hoc* ANOVAs and t-tests separately for Valence (**Figure 2**).

**Table 4:** Statistics for the 3-way ANOVA concerning body-related cognitions.

	*F*	*df*	*P*	*η* *_p_* *²*	Pairwise comparisons for main effects
Group	9.361	1,118	.003*	.073	BED > CG
Condition	535.537	1,118	<.001**	.819	ME > CC
Group × Condition	2.872	1,118	.093	.024	
Valence	195.389	2,236	<.001**	.623	negative > neutral > positive
Group × Valence	34.536	2,236	<.001**	.226	
Valence × Condition	93.652	2,236	<.001**	.442	
Group × Condition × Valence	14.519	2,236	<.001**	.110	

BED, females with binge eating disorder; CC, control condition; ME, mirror exposure condition; CG, females with overweight and obesity. * p < .05; ** p < .001

For negative, neutral, and positive cognitions, significant main effects for Group and Condition as well as significant interactions between Group × Condition were found [all *F*s(1,118) *≥* 6.465, *p*s ≤ .012, *η* *_p_* *²*s ≥ .052]. In both conditions, women with BED verbalized more negative body-related cognitions than the CG. However, this was more pronounced during ME than CC [ME: *t*(118) = 6.182, *p* < .001; CC: *t*(118) = 3.893, *p* < .01]. Concerning neutral body-related cognitions, no difference was found between the two groups in the CC, while during ME fewer neutral body-related cognitions were verbalized by women with BED compared to the CG [ME: *t*(118) = 4.370, *p* < .001; CC: *t*(118) = .932, *p* = .353]. The same pattern emerged for positive body-related cognitions. While no difference was found during CC, significant fewer positive body-related cognitions were verbalized by women with BED compared to weight-matched controls during ME [ME: *t*(118) = 3.707, *p* < .001; CC: *t*(118) = .870, *p* = .386].

### Subjective Ratings of Stress, Insecurity, and Body Satisfaction

*Stress:* Regarding subjective stress level during the thought-sampling procedure, a 2 (Group: BED, CG) × 3 (Condition: Baseline, CC, ME) repeated-measures ANOVA revealed significant main effects of Group [*F*(1,118) = 27.557, *p* < .001, *η* *_p_* *²* = .189] and Condition [*F*(2,236) = 24.701, *p* < .001, *η* *_p_* *²* = .173] as well as a significant interaction of Group × Condition [*F*(2,236) = 3.686, *p* = .027, *η* *_p_* *²* = .030]. While there was no difference between baseline and CC in either group (all *t*s(59) ≥ 1.055, *p*s ≥.296), both groups showed a significant increase in subjectively rated stress level between CC and ME [BED: *t*(59) = 6.038, *p* < .001; CG: *t*(59) = 3.556, *p* = .002]. The increase between baseline and ME was only significant for women with BED [BED: *t*(59) = 4.562, *p* < .001; CG: *t*(59) = 2.288, *p* = .078]. Separated for groups, women with BED reported significantly stronger feelings of stress in all conditions being most pronounced during ME [all *Fs*(1, 118) ≥ 13.298, *ps* ≤.001, *η* *_p_* *²*s ≥ .101].

*Insecurity:* There was a significant main effect of Condition [*F*(2,236) = 35.367, *p* < .001, *η* *_p_* *²* = .231] with higher levels of insecurity during ME compared to CC and baseline [all *t*s(119) ≥ 5.548, *p*s ≤ .001], while there was no difference between insecurity ratings during baseline and CC [*t*(119) = 2.173, *p* = .095]. There was further a significant main effect of Group [*F*(1,118) = 24.579, *p* < .001, *η* *_p_* *²* = .172], whereby women with BED reported higher levels of insecurity compared to the CG. The interaction of Group × Condition was not significant [*F*(2,236) = 2.009, *p* = .136, *η* *_p_* *²* = .017].

**Figure 3:**
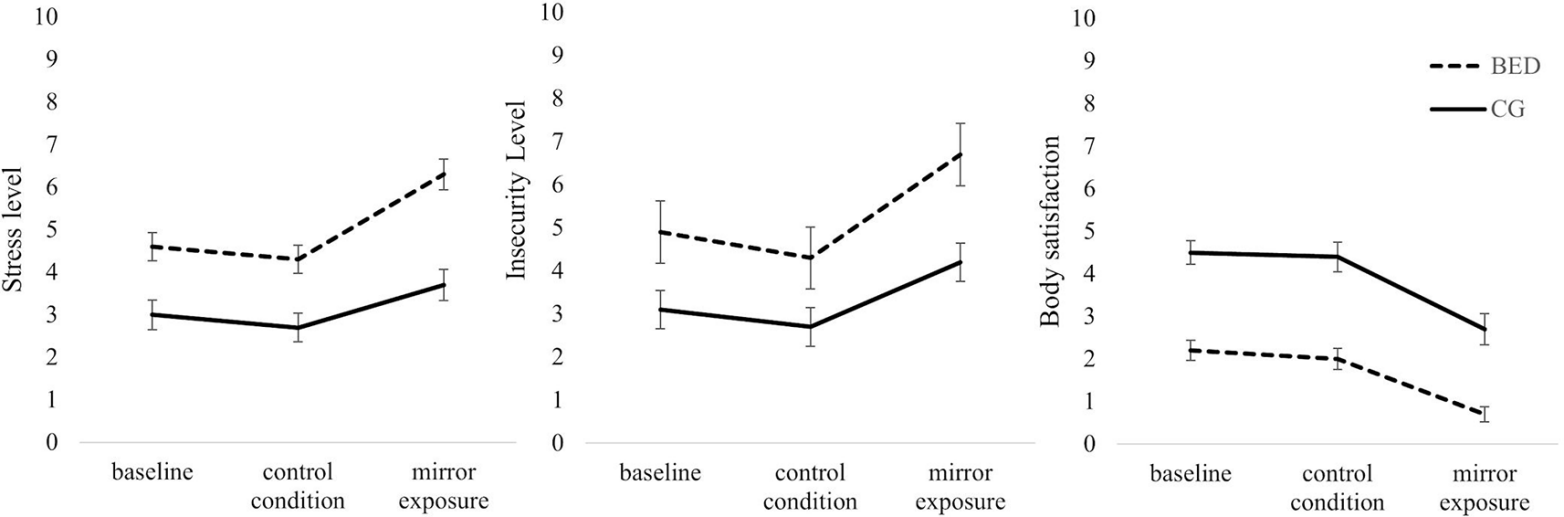
Mean and standard error for self-reported levels of stress, insecurity and body satisfaction at the beginning of the experiment (baseline) and after the control and mirror exposure condition in women with binge eating disorder (BED) and women with overweight and obesity (CG). (Order of the two experimental conditions was counterbalanced between groups. Therefore, the figure presented does not display time sequence).

*Body satisfaction:* There was a significant main effect of Condition [*F*(2,234) = 49.208, *p* < .001, *η* *_p_* *²* = .296]. Thereby, body satisfaction ratings were significantly lower during ME compared to baseline [*t*(118) = 8.341, *p* < .001] and CC [*t*(111) = 8.581, *p* < .001], while no differences between baseline and CC were found [*t*(118) = .771, *p* = .442]. There was further a significant main effect of Group [*F*(1,117) = 52.217, *p* < .001, *η* *_p_* *²* = .309] with the BED group reporting lower ratings of body satisfaction. The interaction of Group × Condition was not significant [*F*(2,234) = .778, *p* = .458, *η* *_p_* *²* = .007] (see **Figure 3** for all VAS).

### Correlations With Fundamental Frequency

No significant correlation was found between the difference score of fundamental frequency and negative body-related cognitions during ME as well as with subjective ratings of stress and body satisfaction during ME (all *r*s ≤ -.145, p ≥.118). However, there was a significant negative correlation between fundamental frequency and the BSQ (*r* = -.189, *p* = .041).

## Discussion

The aim of the present study was to investigate psychophysiological and cognitive-affective responses during an experimental mirror exposure in women with BED by means of vocally encoded emotional arousal. For this purpose, women with BED and weight-matched controls without an eating disorder underwent an experimental mirror exposure as well as a control condition by implementation of a thought-sampling procedure in a repeated-measures design. F0 was used to assess physiological activation as it has already been implemented as an index of emotional arousal during stress-provoking tasks in other mental disorders. In addition, vocal arousal is non-invasive and invisible to participants, and may therefore be better suited as a physiological assessment method relative to more distracting, visible measurements (38, 56, 57).

In accordance with our hypothesis, a cognitive-affective over-involvement with physical appearance was observed in women with BED compared to weight-matched controls. This was indicated by more negative as well as fewer positive and neutral body-related verbalizations as well as a stronger increase in self-reported stress level between the two conditions in women with BED. The results concerning the quantitative content analysis is in line with previous studies on psychological responses to mirror exposure in women with BED (25, 45). However, in our sample, not only differences in the frequency of negative body-related verbalizations were found, but also significant differences in the amount of neutral and positive body-related cognitions. A possible explanation for this difference might be the weight status of the investigated groups, which was substantially higher in the present sample relative to the Hilbert and Tuschen-Caffier (45) study, in which participants’ BMI was only slightly above normal weight. As body dissatisfaction is increased in women with overweight and obesity (60), and significant differences between normal weight and obese persons with BED have been found (61), this may account for the significant findings relating to positive and neutral body-related cognitions in our study. Furthermore, in line with our results on positive body-related verbalizations, Svaldi et al. (62) found women with BED to be less capable of retrieving positively valenced shape-/weight-related words using an experimental recall task compared to weight-matched controls. Thus, against the backdrop of current cognitive theories of eating disorders, the activation of underlying body-related schemas (63) might not only trigger an enhanced bias for negative body-related cues, but might also impede the retrieval of positive body-related verbalizations, thereby maintaining body dissatisfaction.

In contrast to our hypothesis, the observed cognitive-affective activation in women with BED was not accompanied by increased, but reduced vocally encoded emotional arousal relative to the CG. At the same time, though, in both groups, self-reported levels of stress increased significantly from control to mirror exposure condition. Taking these findings together, a dissociation between self-reported stress level, verbalized body-related cognitions, and objectively measured physiological distress by means of f0 was found in women with BED. This was further supported by an inverse correlation between severity of self-reported body dissatisfaction and f0.

In line with previous studies, blunted physiological reactions to psychosocial stressors have been found in women with BED (33, 64) as well as in other non-clinical and eating-disordered samples during mirror exposure (e.g., 29, 31). The reason for these blunted reactions, however, is poorly understood. General changes in the psychophysiological response capability in eating disorders due to starvation and intermittent dieting are discussed (e.g., 65). Furthermore, habituation processes might be able to explain altered responses referring to the fact that females with BED are so used to these negative cognitions and emotions about their own body, that these cognitive-affective reactions do not elicit a response anymore. To clarify the specific mechanisms underlying these blunted responses, further studies are needed. So far, this dissociation between cognitive-affective and physiological reactions to body-related distress has been interpreted as an inability of body-dissatisfied persons to adaptively engage in physiological responses to body-related distress. This interpretation is based on the concept of different emotion theories (e.g., 66, 67). Accordingly, emotional coherence between experiential, physiological, and behavioral reactions helps individuals to effectively cope with environmental demands, while a lack of emotional coherence might reflect emotional dysregulation. As emotion regulation problems are a well-known phenomenon in women with BED (68, 69), the smaller increase in f0 between the two experimental conditions in participants with BED relative to controls may be attributable to their previously reported emotion regulation difficulties. This is in line with a study in which emotional coherence after a negative, positive, and neutral mood induction was stronger in response to a more functional relative to a more dysfunctional emotion regulation strategy (70). In future studies, this variable should therefore be additionally assessed when investigating the psychophysiological effects of mirror exposure.

Alternatively, the lack of coherence found in the BED group might also be attributable to the particular emotions activated during mirror exposure. Studies which have investigated emotions during mirror exposure found women with BED and BN to react with increased levels of sadness, insecurity, tension, anxiety, and disgust (25, 26). Note that for vocally encoded emotional arousal, an *increase* in mean f0 has been found for anger, joy, fear, anxiety, and stress, while a *reduction* in mean f0 has been associated with sadness, disgust, contempt, and boredom (36). Thus, our induction may have activated counteracting emotions in women with BED, which may account for the smaller increase in f0. It is important to note that not only f0 is prone to this limitation, but also other physiological assessment methods (71, 72). This might furthermore explain why associations between vocally encoded emotional arousal and the self-reported experience of stress and anxiety was more consistently found in previous studies in anxiety disorders (38, 57). Taken together, these findings point to the importance of investigating more thoroughly the different emotions activated through mirror exposure as it might be a confounding variable.

Against the backdrop of the adverse effects of a negative body image in women with BED (11, 16, 17), some important conclusions can be drawn from the present study. The high negativity of body-related cognitions during mirror exposure in the present BED sample might directly maintain binge eating, as a recent study has shown, that the negative influence of body shame on binge eating is directly mediated by enhanced self-criticism (73). Therefore, body-related interventions in BED should focus on adaptive strategies to enable a better regulation of body-related distress during exposure like displayed by the present CG. While mirror exposure has shown to be effective for the improvement of the cognitive-affective component of body image in BED (23, 24), the specific underlying mechanisms are not yet understood (19). Future studies should therefore focus on mechanisms of change which account for the observed improvements. A recent study conducted on stress-induced changes in state body dissatisfaction in women with BED found the cognitive-affective (anxiety, tension, distress, urge to leave the situation) rather than the biological stress response to be predictive for changes in body dissatisfaction (33). This underpins the importance of cognitive-affective changes during mirror exposure. However, the results on vocally encoded emotional arousal should not be neglected. Albeit unexpected, the observed blunted physiological reaction during mirror exposure in women with BED might be of predictive value for treatment outcome given the significant negative correlation with self-reported body-satisfaction. As the predictive validity of f0 has already been proven in pharmacological and psychotherapeutic treatment in other mental disorders (34, 44), future studies should focus on clarifying this relationship.

The present results should however be interpreted considering some limitations. First, the control condition used in the present study needs to be reconsidered. As a higher stress level was observed in the BED group across the two experimental conditions, future studies should implement a more suitable control condition. This notwithstanding, the anticipatory activation found in the BED group corroborates other studies on physiological arousal in eating and non-eating disordered women high on body dissatisfaction (e.g., 31, 33). In the case of the present study, this might have been because participants were informed that they will undergo a mirror exposure within the experimental session. Therefore, future studies should use authorized deception in order to avoid the activation of body-related schemas in the control condition (with the disadvantage not to be able to randomize the experimental sequence of the control and mirror exposure condition). This is important though, as the heightened stress level prior to the exposure might adversely influence vocally encoded emotional arousal. In fact, a recent study (74) found that a specific level of emotional response (in this case by means of cortisol) was necessary to observe vocal changes in reaction to stress in healthy controls. Therefore, heightened self-reported stress-levels prior to mirror exposure might have accounted for the smaller difference score in f0 in women with BED. In other words, changes in vocal arousal might have already appeared in the anticipatory control condition.

Second, even though we used a large sample with an age and weight-matched control group, no men were included in the study. As gender is said to influence body dissatisfaction (75), studies including men should be used to further understand physiological arousal during mirror exposure. Third, the coding system used in this study was only able to capture differences in frequency, but not in the intensity of body-related cognitions. That is, sentences such as “I don’t like my body” compared to “I really hate my body” were both coded as one negative body-related statement. Future studies should refine this coding system including a rating on intensity. Fourth, the VAS scales used to assess insecurity, stress, and body satisfaction over the course of the experiment might be limited in terms of their reliability. Future studies should focus on including validated questionnaires to assess negative emotions. Of note, as stated above, the differentiation between different negative emotions seems to be especially relevant in the context of investigating physiological reactions as different emotions might differentially influence these measures. Finally, there is some evidence that different physiological systems (e.g., parasympathetic, sympathetic, endocrinological system) might react dissimilar during stressful demands. In two studies on mental stress in women with BED, different changes were found for the different physiological markers used (76, 77). Hence, there is a need to include different physiological assessment methods in future studies.

To conclude, this was the first study to assess physiological arousal during mirror exposure in women with BED compared to weight-matched controls by means of vocally encoded emotional arousal. While findings on cognitive-affective over-involvement during mirror exposure were replicated, surprisingly, no coherence emerged in terms of vocally encoded emotional arousal. This might be explained by an inability of women with BED to create adequate physiological responses to body-related distress, which might adversely influence emotion regulation during confrontation. However, this interpretation should be treated with caution as a dissociation of emotional coherence might not necessarily reflect a dysregulated system (78). For this reason, future studies should take into account different physiological assessment methods besides investigating emotions and emotion regulation strategies activated during mirror exposure to control for potential confounding variables.

## Data Availability Statement

The datasets generated for this study are available on request to the corresponding author.

## Ethics Statement

The studies involving human participants were reviewed and approved by the Ethics Committee of the Medical Faculty of the University of Tuebingen. The patients/participants provided their written informed consent to participate in this study.

## Author Contributions

JB, KK, BT-C, and JS contributed to conception and design of the study. JB and KK were responsible for data collection. JB and JS performed statistical analysis and wrote the first draft of the manuscript. KK, BT-C, and JS contributed to manuscript revision and mentoring.

## Funding

The study was partially funded by the Deutsche Forschungsgemeinschaft (DFG, German Research Foundation) accorded by Prof. Dr. Jennifer Svaldi and Prof. Dr. Brunna Tuschen-Caffier (SV83/3-1). The article processing charge was funded by the Deutsche Forschungsgemeinschaft (German Research Foundation; DFG) and the Eberhard Karls University Tuebingen in the funding program Open Access Publishing.

## Conflict of Interest

The authors declare that the research was conducted in the absence of any commercial or financial relationships that could be construed as a potential conflict of interest.
